# Supplementary material for: Defining new Buruli ulcer endemic areas in urban southeastern Australia using bacterial genomics-informed possum excreta surveys
Source: Appl Environ Microbiol. 2025 Nov 18;91(12):e01602-25. doi: 10.1128/aem.01602-25 (PMC12724319; doi:10.1128/aem.01602-25)
Supplement: Figure S1 — Distribution of M. ulcerans IS2404 qPCR cycle threshold values across surveys. [file aem.01602-25-s0002.pdf]

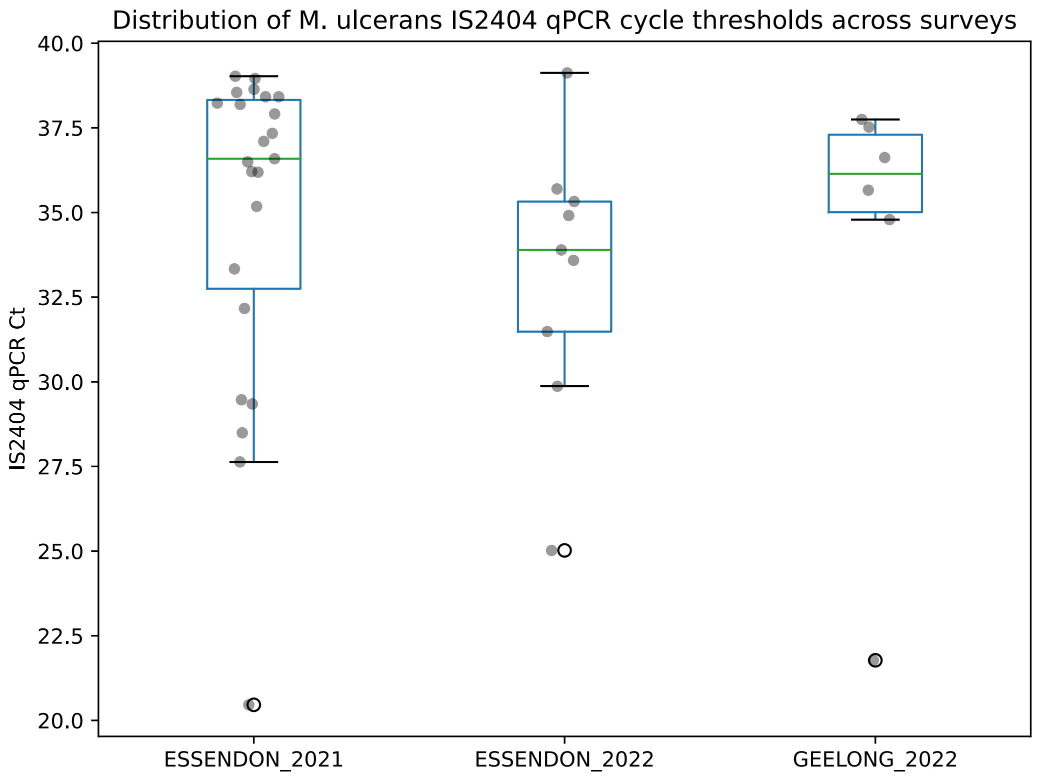

Figure S1. Distribution of *M. ulcerans* IS2404 qPCR cycle threshold (Ct) values across surveys. Boxplots show the interquartile range, median, and whiskers (1.5×IQR) for positive possum excreta samples collected in Melbourne's inner north in 2021 and 2022 and in Geelong in 2022. All individual data points are overlaid on the boxplots.
